# What About the Men? A Critical Review of Men’s Experiences of Intimate Partner Violence

**DOI:** 10.1177/15248380211043827

**Published:** 2022-01-30

**Authors:** Kelly Scott-Storey, Sue O’Donnell, Marilyn Ford-Gilboe, Colleen Varcoe, Nadine Wathen, Jeannie Malcolm, Charlene Vincent

**Affiliations:** 1 Faculty of Nursing, University of New Brunswick, Fredericton, NB, Canada; 2 Arthur Labatt Family School of Nursing, 70383 University of Western Ontario, London, ON, Canada; 3 School of Nursing, 70439 University of British Columbia, Vancouver, BC, Canada

**Keywords:** men, intimate partner violence, gender, men’s health, measurement

## Abstract

Intimate partner violence (IPV) is a health problem affecting people of all genders and other social locations. While IPV victimization of cis-gendered women has been widely researched, how men conceptualized or experience IPV victimization, and the variations in their experiences of IPV, has not been thoroughly examined. In this critical review of men’s experiences of IPV, an extensive search of peer reviewed literature was conducted using multiple database (Cochrane database, MEDLINE, CINAHL, Embase, PsycgINFO, and Google Scholar) as well as the gray literature. We critically reviewed examining the conceptual foundations of IPV victimization among men. The influence or gender roles and societal expectation on men’s experiences and perceptions of IPV victimization and their help-seeking behavior are explored. Current knowledge about types, tactics, and patterns of IPV against men and the health and social consequences of IPV are addresses. Additionally, the conceptual and empirical limitations of current research are discussed, including the tendency to compare only the prevalence rates of discrete incidents of abuse among women versus men; the use of IPV measures not designed to capture men’s conceptualizations of IPV; and the lack of attention given to sex and gender identity of both the victim and perpetrator. Future research priorities that address these limitations and seek to strengthen and deepen knowledge about IPV among men are identified.

## What About the Men? A Critical Review of Men’s Experiences of Intimate Partner Violence

This critical review of the literature explores how men understand and conceptualize experiences of intimate partner violence (IPV) victimization, and examines current knowledge about the gendered types, tactics, patterns, and impacts of IPV victimization in men. A key goal is to identify conceptual and empirical gaps related to IPV experienced by men and related research priorities. IPV is a serious public health problem that results in significant social and economic costs (Haegerich & Dahlberg, 2011; Varcoe et al., 2011; World Health Organization (WHO), 2016; Zhang, et al., 2012). It is defined as any behavior within an intimate relationship (past or current) that causes or has the potential to cause physical, sexual, or psychological harm, including acts of physical aggression, sexual coercion, psychological abuse, and controlling behaviors, including financial abuse (WHO, 2010). IPV affects people of every race, class, age, socioeconomic status, gender, sexual identity, and relationship status (Renner & Whitney, 2010).

Globally, IPV has been recognized as a gendered issue, disproportionately affecting women (WHO, 2010). Therefore, much of what is currently known about IPV comes from samples of women and, in particular, cisgender heterosexual women in relationships with men. Although there is general consensus that men are also victims of IPV, as described above (Hines, 2015; Perryman & Appleton, 2016), relatively few studies have explored IPV against men (Arnocky & Vaillancourt, 2014; Cook, 2009; Hines & Douglas, 2010; Migliaccio, 2001; Perryman & Appleton, 2016; Tilbrook et al., 2010). Even less attention has focused on variation in experiences of IPV *among* men, for example, among gender and sexual minority men (Randle & Graham, 2011; Rollè et al., 2018). The result is an inadequate understanding of how IPV is experienced by men across varied categories of difference (Cook, 2009; Hines & Douglas, 2010; Migliaccio, 2001; Perryman & Appleton, 2016; Tilbrook et al., 2010).

Because of a limited focus on men’s experiences, how men define or conceptualize violence continues to be poorly understood (McHugh et al., 2013) and, thus, such perspectives may not be clearly reflected in measures of IPV. As a result, measures that were developed for use among women have been used with men without critical examination of their validity, applicability, and fit (Finneran & Stephenson, 2012). For example, the Women’s Experience with Battering Scale (WEB) which was developed for use among women, has been used to draw conclusions about sex differences between women and men who were both victims and perpetrators of IPV (Houry et al., 2008). Importantly, McHugh and colleagues (2013) caution that simply changing the pronouns on a scale without completing validity testing is inadequate for understanding how men view and interpret violence victimization. Therefore, whether many of the commonly used IPV scales are appropriate for use with men remains unknown.

As a foundation for defining and measuring men’s experiences of IPV, there is a need to consider some critical questions: What do men perceive as violent and abusive acts and how do they attribute meaning to these experiences? Are the types, tactics, patterns, and consequences of IPV that men experience similar to or different from those experienced by women? Are there other important factors that influence men’s experiences of violence that ought to be considered (i.e., factors that account for variation in IPV experiences such as race, class, gender, and sexual identity)? As a caveat, we use the term “men” to represent individuals who self-identify as men. However, heteronormative biases exist in current evidence; therefore, much research has been based on the assumption that men represent an undifferentiated category of cisgender heterosexual men. While we recognize the importance of understanding and addressing experiences of IPV among *all* men, many papers reviewed do not take gender identity or sexual orientation into account and/or tend to compare men to women, rather than examining variations in experiences among men*.* This limitation is evident in our review and has implications for future research. As such, we address the role of gender in the context of evidence about men’s experiences of IPV.

## Method

In undertaking this critical literature review of the current state of knowledge of men’s experiences of IPV, the authors were interested in reviewing a broad scope of literature and evidence sources, and therefore, we did not apply search criteria commonly used in systematic reviews, meta-analyses and scoping reviews. Rather, we conducted an extensive search of peer reviewed qualitative, quantitative and mixed-method manuscripts, government documents, theoretical papers, dissertations, reviews and commentaries, and gray literature across multiple databases (Cochrane, MEDLINE, CINAHL, Embase, PsychINFO, and Google Scholar) and relevant government and agency websites. We also used a “snowballing” approach that involved reviewing reference lists of relevant articles to identify additional literature. Literature was limited to English only; no date restrictions were used as it was important to explore developments in understanding and trends overtime. Key search terms included “intimate partner violence and men,” “domestic violence and men,” “partner violence and men,” “male victims of violence,” and “men as victims of violence”; variations of these terms were used to ensure exhaustive search results. We adopted a critical perspective to evaluate and synthesize the literature; our approach did not produce a quantitative account of articles as this was not the intent given our purpose to broadly understand men’s experiences of violence victimization.

### Men’s Experiences of Intimate Partner violence

#### Role of gender

Intentional consideration of the role that gender and masculinities play in men’s IPV experiences is required. It should be noted that we view gender as a social construct that continually shifts overtime in relation to social and historical contexts (Connell, 1995; 2005). The term masculinities reflects the socially constructed nature and variation in the ways that gender is constructed and expressed among men (Connell, 1995; 2005). According to Nybergh et al. (2016), it cannot be assumed that violence is viewed and experienced in similar ways by men and women since “gender as a pervasive structure affects both expression and experiences of IPV” (p. 199). To extend this, gender not only accounts for important differences related to views, expressions and experiences of violence between women and men, but for variation *among* men. Views about what it means to be a man and social expectations related to gender influence not only constructions and expressions of violence, but also how men define, label (Allen-Collinson, 2009; Hamberger & Guse, 2002; Holtzworth-Munroe, 2005) and respond to IPV (Cook, 2009; Hamberger & Guse, 2002).

Although varying constructions and expressions of gender exist, generally there are societal expectations of what a “man” should be (Bem, 1981). Western society has been shaped by a patriarchal belief system that positions men as economically, socially and politically dominant (Hines & Malley-Morrison, 2001; Perryman & Appleton, 2016). Connell (1995; 2005) has argued that men are positioned by themselves and others in relation to these ideals with negative and harmful implications for men and their experiences of violence. Not only do men experience social pressure to conform to hegemonic masculine norms (Addis & Mahalik, 2003), deviating from these norms is itself a risk for violence. Men’s reports of being targets of emasculating and homophobic comments in the context of IPV (McHugh et al., 2013; Nybergh et al., 2016), and of violent and abusive behaviors that center around conformity to these ideals, provides evidence that men continue to be positioned by others in relation to dominant gender ideals. Men’s reports of feeling victimized and hurt by such behaviors (McHugh et al., 2013; Nybergh et al., 2016) demonstrate their own positioning in relation to such ideals revealing that, “heterosexual masculinity continues to be collectively constructed by denigrating femininity and homosexuality [*sic*] as ‘not-male’” (Messner, 1994, p. 47).

The harms related to violating gender norms are an important consideration when examining the nature and patterns of violence experienced by men, especially for gay men who experience additional complexities related to their experiences of IPV (Oliffe et al., 2014). Specific additional harms include threats (or acts) to publicly disclose the nature of their romantic relationships (Carvalho et al., 2011), sexuality, and/or to “out” them (Ard & Makadon, 2011; Brown & Herman, 2015; Carvalho et al., 2011). These tactics draw further attention to the powerful and harmful impact of traditional masculine ideals, as seen in rates of IPV in same sex, bisexual, or trans relationships that are comparable to, or higher than, those in heterosexual relationships (Allen-Collinson, 2009; Burke & Follingstad, 1999; Carvalho et al., 2011; Greenwood et al., 2002; McClennen, 2005; Messinger, 2011; Walters et al., 2013; West, 2012).

Such ideals and related expectations also have implications for what men perceive as violent and abusive acts and how they attribute meaning and respond to these experiences. It is theorized that in the context of IPV, pressure to fit with and adhere to dominant gender ideals not only influences men’s sense of self (Morgan et al., 2016), but also their own and others’ appraisal and identification of the violence they experience. For example, in a study of 20 Italian participants, Entilli and Cipolletta (2017) found that when male victims of IPV took responsibility for their female partners’ abusive acts and did not react to physical attacks, they believed they were being a “good partner” and upholding what it meant to be a man. Based on their qualitative study with men who were victims of IPV, Morgan and Wells (2016) suggested that society in general does not endorse the idea that men can be victims of female perpetrated violence. While we cannot ignore that women are disproportionately impacted by IPV (Randle & Graham, 2011), including the most injurious forms, dominant social beliefs and expectations about men as potential victims may influence how men themselves view their victimization. Howard and Hollander (1996) noted the experience of IPV left some men in their study feeling “feminized” (p. 86), reflecting the fact that, in Western society, victimization has been “deeply coded as a female experience” (Allen-Collinson, 2009, p. 35). Eckstein and Cherry (2015) also highlighted the link between gender and appraisal of men’s violence suggesting that men’s victimization by female perpetrators results in a “dual violation of gendered *and* relational expectations” (p. 140). Some researchers have even questioned the use of the term “intimate partner violence” with men, claiming that its significant gendered connotation may impact perceived masculinity (Walker et al., 2019).

Some evidence suggests that when women perpetrate violence against men, it is not always perceived as abusive by men (Hoare & Jansson, 2008; Hogan, 2016; Matte & Lafontaine, 2011). Beyond difficulties in identifying experiences as IPV, when men do identify as victims, gender socialization may lead to a tendency for some men to minimize or trivialize experiences of IPV (e.g., Hamberger & Guse, 2002; Holtzworth-Munroe, 2005). Men not only minimize their experiences, but may also find it difficult to disclose experiences and seek help (Allen-Collinson, 2009; Arnocky & Vaillancourt, 2014; Barrett et al., 2020; Morgan et al., 2016; Tilbrook et al., 2010). Some men have reported avoiding help-seeking for IPV due to fear of gender biased ridicule, shame, or being labeled the initiator of violence (Allen-Collinson, 2009; Brooks et al., 2017; De Puy et al., 2017; Gaman et al., 2016; Hines & Douglas, 2009; Machado, et al., 2017; McNeely et al., 2001; Tilbrook et al., 2010; Walker et al., 2019). For example, in a study of 258 men who had experienced IPV by a female partner, Walker et al., (2019) found that police exhibited gender-biases, by accusing male victims of being the perpetrators of the violence and threatening them with arrest. The consequences of these perceptions and actions can be significant, as gender stigmatization that impedes men from showing emotional vulnerability, disclosing abuse/violence or seeking help (Hines, 2015) means that health and other challenges related to violence may go unaddressed.

Even when men do disclose IPV, they may not get the support they need. Morgan and Wells (2016) reported that, when attempting to seek help and/or services for IPV, some men described that they were not taken seriously. Based on their qualitative work, Brooks et al. (2017) also noted that some of the men in their study expressed concerns about being doubted by others and denied assistance; as one man noted, “guys don’t call the police . . .because they don’t do anything about it anyway” (p. 13). Sexual minority and non-cisgender people experiencing IPV face even greater social stigma, including homophobia and transphobia, further contributing to the complexities in understanding and addressing men’s experiences of IPV (Carvalho et al., 2011; Lewis et al., 2012; Meyer, 2012; Oliffe et al., 2014; Perryman & Appleton, 2016).

### Types, Tactics and Patterns of Intimate Partner Violence Among Men

Men who are victims of IPV experience a broad array of abuse types and tactics. In a study of 302 men seeking IPV services, Hines and Douglas (2015) reported that men were victims of a range of physical, psychological, and sexual violence, and abusive and controlling tactics consistent with the WHO definition of IPV (WHO, 2010) and in line with those typically identified by women. McHugh (2005) and Tilbrook et al. (2010) also noted similarities in the types of violence experienced by men and women.

While these reports provide evidence that men experience a range of types of violence, and broadly establish IPV as an important health and social issue for men, we caution that comparing women and men without considering broader social and structural factors that contribute to power and vulnerability can result in harm, such as by minimizing the severity of violence within specific groups. Thus, our aim is not to complete a comparative analysis between men and women but to examine men’s reports of IPV while considering factors that account for variation *among* men. This includes examining evidence about the types of IPV men experience, as well as descriptions of the nature, severity, and variations in patterns of these experiences.

### Physical Violence

Consistent with the WHO (2010) definition, acts of physical violence include slapping, hitting, kicking, and beating. In the context of IPV, men report having been pushed, shoved, grabbed, shaken, slapped, hit, kicked, bitten, scratched and/or threatened or harmed with a knife or other object (Brooks et al., 2017; Carmo et al., 2011; Drijber et al., 2013; Gadd et al., 2003; Hines, 2015; Machado et al., 2018; Nybergh et al., 2016; Savall et al., 2017). One man in Brooks et al.’s (2017) study noted, for example, “She was hitting me in the face area . . . with her fists and like I said, she’s not weak . . . ” (p. 8) while another described, “I just let her dig her claws into me, I didn’t even fight back. I just want to see my kids . . . ” (p. 11). Some researchers report that women may launch physical attacks when the man is unable to retaliate, such as from behind, when the man was sleeping or when children were present (Bates, 2020; Entilli & Cipolletta, 2017; Hogan, 2016; Walker et al., 2019). Unfortunately, these reports are generally decontextualized from frequency, pattern, and impact, making it challenging to fully understand the experience of physical violence for men.

In their qualitative study, Nybergh et al. (2016) noted that while physical violence was often used by female partners, it was rarely perceived as a tactic effective for controlling the man. Rather, most men described feeling in control of their female partners’ physical aggression and able to stop it by “walking away, holding them back, or retaliating” (p. 196). De Puy et al. (2017) also noted that men sometimes physically restrained their partners when they were being violent. Thus, on the whole, men seldom interpreted their female partners’ physical violence as serious, intimidating, frightening, or posing a genuine threat (Anderson, 2005; Swan & Snow, 2006). However, variation in men’s experiences does exist and some men do report fear related to physical violence. One man in the Brooks et al. (2017) study shared, “I had fears big time because of how vicious this woman is, you know I would have fears . . . she could be stalking me, that kind of fear. She could be conjuring up something for my failure” (p. 8). In other studies, men have reported fear related to threats of physical violence, sometimes directed at their family (Bates, 2020; Walker et al., 2019). While the perception of danger/severity and related distress are important, how common these experiences are for men and whether they represent the norm, or an extreme, is poorly understood.

In their critical review of the literature examining IPV among heterosexual and gay men, Nowinski and Bowen (2012) noted that gay men were more likely to experience similar or higher rates of physical violence than heterosexual men. Nybergh et al. (2016) identified that some men experience fear and terror, for example, one gay man in their study described being “terrified” due to their partner’s physical strength and the physical harm he suffered. Adding to the complexity of understanding men’s experiences of physical IPV, others have noted more reciprocity or bi-directionality of physical violence among men who are in relationships with men compared to men in relationships with women (Stanley et al., 2006). These findings highlight that physical violence may give rise to varying responses among men and emphasize the importance of not only focusing on the occurrence of physically violent acts, but what they invoke (i.e., how distressing or fear-inducing a particular act, threat or experience of physical violence is), as well as the need to capture the sex and gender of perpetrators in studies of IPV among men.

### Sexual Violence

Varied definitions of sexual violence used in research can contribute to confusion in understanding prevalence rates and experiences of sexual violence among men. When sexual violence is narrowly defined as “rape” or “forced sex” (which implies physical force), heterosexual men in relationships with women may be less likely to identify themselves as victims. This may be due to differences in physical anatomy, size, and strength as some have reported that women may not be physically capable of achieving this level of sexual power over men (Ferraro, 2013; Follingstad & Rogers, 2013; Swan, et al., 2008; Tanha, et al., 2010). While being physically “forced” into sex or sex acts may remain gendered in heterosexual relationships (i.e., women are more likely to be victimized by male partners), when broader definitions of sexual violence are used, different prevalence rates for men emerge. For example, men have reported being coerced or pressured by their partners to engage in unwanted sexual acts and/or activities or unprotected sex through the use of threats, manipulation, pressuring, and false promises (Bates, 2020; Follingstad & Rogers, 2013; Machado et al., 2018; Struckman-Johnson & Struckman-Johnson, 1998; Walker et al., 2019). Among men, forced sex and sexual violence is more prevalent in relationships with other men than in relationships with women (Nowinski & Bowen, 2012) and gay and bisexual men are more likely to report sexual violence than heterosexual men (Dickerson-Amaya & Coston, 2019). Further, there is some evidence to suggest that HIV-related sexual violence, such as not disclosing HIV status or deliberately transmitting HIV, is a tactic used by men in relationships with men (Stephenson & Finneran, 2017). The emerging differences for men in relationships with other men emphasizes the need for measures that ask about partner sex and gender, and capture acts of sexual violence beyond those using physical force.

### Psychological Violence including Coercive Control

There is growing evidence to suggest that psychological violence may be the most common form of IPV experienced by men (Follingstad & Rogers, 2013). One Canadian study, using data from a national survey, reported 10.1% of men reported experiencing at least one type of psychological and/or economic abuse from their current partner (Dim & Elabor-Idemudia, 2018). Psychological, mental, and emotional violence are terms often used interchangeably to mean acts, threats, or coercive tactics intended to humiliate, degrade, or undermine a person’s self-worth or self-esteem, to control, and/or isolate (Breiding et al., 2015; WHO, 2012). Examples of psychological violence include “insults, belittling, constant humiliation, intimidation (e.g., destroy things), threats of harm [*and*] threats to take away children” (WHO, 2012, p. 1). While there is not yet a thorough understanding of how men define psychological violence (McHugh et al., 2013), in some studies men have described being yelled at, insulted, belittled, humiliated, having their sexuality questioned, being controlled, monitored, isolated from family and friends, having their competence as a father questioned, false allegations of child abuse, and enduring threats of having their children taken away (e.g., Allen-Collinson, 2009; Bates, 2019, 2020; Entilli & Cipolletta, 2017; Follingstad, 2007; Machado et al., 2018; McHugh et al., 2013; Nybergh et al., 2016; Tillbrook et al., 2010; Walker, et al., 2019). In Tillbrook et al.’s (2010) qualitative study, for example, one man described feeling degraded both personally and as a father, “running me down both as a person and as a dad . . . literally just pick things to bits she would criticize things that I would do personally . . . telling me I was the most useless piece of shit that she had ever seen” (p. 17).

Men report feeling monitored and controlled (Nybergh et al., 2016) and contemplating or attempting suicide in response to the violence (Bates, 2019; Machado et al., 2017).

There is evidence to suggest that psychological tactics including insults and threats directed towards men by women may be different than how men tend to insult or threaten women; for example, Matte and Lafontaine (2011) suggested that men do not readily identify with being called “fat” or “ugly,” a tactic commonly reported by women. Some men report being ridiculed and belittled for not being “manly” enough, not making enough money, being weak, or for crying when hit (Bates, 2019; Nybergh et al., 2016). Emasculating comments and homophobic language are tactics identified by men as being particularly controlling and hurtful (McHugh et al., 2013; Nybergh et al., 2016). This is consistent with what McHugh et al. (2013) describe as “gender role harassment,” a form of psychological abuse that may be more commonly used against men (p. 168). While most men do not fear physical violence from a female partner, some studies find they fear degradation and humiliation, especially in public (Bates, 2020; Nybergh et al., 2016). Research in this area remains in its infancy and further investigation into men’s accounts of psychological violence and the intersections with gender is required.

Within IPV research, coercive control has been identified as important in explaining why some perpetrators use violence and also how this type of violence impacts victims. When power and control motivates violence between partners, victims are subjected to more severe violence with more damaging effects on physical and mental health (Whitaker, 2013). While men do report being controlled by female partners, it is rarely by means of physical aggression; rather, men feel controlled through their partners’ use of children (i.e., feeling trapped in the relationship or fear of losing custody or access), fear of becoming socially isolated, being monitored/restricted in activities, through false accusations of abuse (towards partner or children), blackmail, and manipulating behaviors (Bates, 2020; Corbally, 2015; Drijber et al., 2013; Hines & Douglas, 2010; McHugh et al., 2013; Morgan & Wells, 2016; Tilbrook et al., 2010; Walker et al., 2019).

Economic and financial abuse is another means of control reported by men, for example, having restricted or no access to bank accounts or spending money (Tsui, 2014; Walker et al., 2019). Legal and administrative abuse has also been identified as a mechanism of control that men experience from female partners (Hines et al., 2014; Machado et al., 2017; Tilbrook et al., 2010). This occurs when legal or administrative systems are used to harm one’s partner, and can encompass some of the tactics mentioned above. Such forms of abuse can have potentially devastating consequences, including loss of child custody and financial instability (Hines et al., 2014). What remains unclear are the impacts of coercive control for men and how these might be unique to or similar among men.

### Patterns of Intimate Partner Violence

While the overview above deals with the various types of IPV separately, victimization for both men and women often includes more than one type of violence concurrently (Scott-Storey, 2011). Men have reported a range of IPV experiences, from isolated incidents to repeated patterns of severe violence, intimidation, and control (Hines & Douglas, 2010; Tilbrook et al., 2010). Results from qualitative research revealed that men experience episodes of abuse that start with less violent acts early in the relationship and then escalate into more severe forms of violence, which intensified with life and/or relationship changes, such as the birth of a child (Entilli & Cipolletta, 2017; Machado et al., 2017). While these studies point to commonalities between men and women in relation to the overall types experienced, some differences in *patterns* of violence have been identified.

In their latent class analysis of data from a large Canadian population-based survey (N = 7056 men and 8360 women), Ansara and Hindin (2010) identified patterns of IPV among men and women based on modified self-reported questions from the Conflict Tactics Scale (Straus, 1979). Six patterns of IPV were identified for women and four patterns for men. Three of these patterns (no abuse, jealousy and verbal abuse, and physical aggression) were common to both women and men but there were also differences in patterns of IPV between men and women. For example, women were classified as experiencing *severe* violence, control, and verbal abuse whereas men were classified as experiencing *moderate* violence, control, and verbal abuse. Further, women experienced distinct patterns of IPV that men did not (e.g., physical aggression, control, and verbal abuse). Ansara and Hindin (2010) reported that the most common IPV pattern among men did not include physical violence, but was characterized by jealousy and verbal abuse. Furthermore, very few men reported violence in the form of unwanted sexual activity. This study extends the results of qualitative research by confirming that men experience physical, sexual, and psychological IPV but that patterns of IPV that represent serious physical and sexual violence tend to be predominately experienced by women. While the results of this study are important, a limitation is that disaggregated patterns identified for women and men based on the gender and sex of the partner were not reported. Whether patterns of violence differ depending on the gender and sex of the partner is poorly understood and requires further inquiry.

Embedded in the discussion of abuse patterns, recent theoretical and empirical work suggests there are distinct subtypes of IPV based on characteristics of both the victim and perpetrator and that prevalence rates depend on which subtypes are being examined (Anderson, 2005; Ansara & Hindin, 2011; Johnson, 2011). Johnson (2006) asserted that within any relationship, repeated acts of violence entrenched in tactics of coercive control and intimidation, and which are used to elicit fear and terror, reflect a specific subtype of IPV known as “intimate terrorism.” Intimate terrorism is reinforced by patterned threats and/or acts of violence that often escalate in frequency and severity over time and result in the most severe health and social consequences for the victim (H. Johnson, 2015). Intimate terrorism is predominantly perpetrated by men, although not uniquely (M. Johnson, 2011). Using national Canadian survey data, Laroche (2005) concluded that most victims, both male and female, who suffered serious physical and psychological consequences were categorized as being victims of intimate terrorism, but that the prevalence was significantly higher among women than men (249,400 vs. 138,000). Hines and Douglas (2018) found that men experiencing intimate terrorism reported more symptoms of depression, PTSD, higher rates of problematic physical health symptoms (such as pain and fatigue) and injuries than men in a population-based sample. As a further example of variation in subtypes of violence, “situational couple violence” has been described as IPV that is comprised of occasional, mutual, and lower intensity acts that reflect attempts to deal with conflict, rather than to exert power and control over the partner through use of fear and intimidation; it has been reported that this type of violence is perpetrated almost equally by men and women (M. Johnson, 2006, 2008; McHugh, 2005). Violent resistance and mutual violent control subtypes, while less common, may also have explanatory value for men’s experiences of violence; more research is needed in this area. However, emerging evidence suggests that these subtypes occur along a continuum from less to more severe rather than distinct categories (Love et al., 2020).

Identifying and examining different patterns of IPV offers promise in understanding how gender shapes experiences and consequences of IPV, with implications for tailoring interventions and services. However, most IPV measures identify particular acts of violence at specific points in time, or over a defined period (e.g., past 1 year and past 5 years). This makes it difficult to assess trajectories of violence over time, including shifts in the severity of abuse, and the context or effect of terror and fear, and thus, the distinction of unique “subtypes” of violence (i.e., intimate terrorism vs. situational couple violence) (Kelly & Johnson, 2008; Johnson, 2008). The majority of measures, such as the Conflict Tactics Scale, used in large-scale surveys primarily capture situational couple violence and not intimate terrorism (M. Johnson, 2006; Johnson, 2015), leading to inadequate understanding of the nature and prevalence of more serious forms and patterns of IPV among men and women.

Reed (2008) called attention to concerns related to measurement as limiting description of patterns, and argued that instruments that only assess whether a particular act of violence (e.g., hitting) is experienced (yes/no) “lack specificity to capture other core elements of IPV (e.g., control, patterning of abuse, intimidation)” (p. 198). Próspero (2007) also highlighted the limitations of merely focusing on acts of violence suggesting that, “this view assumes that a slap or a push by a man is equivalent to a slap or a push by a woman and fails to measure the different consequences of those acts, which may reveal gender asymmetry in IPV outcomes” (p. 270).

Newer measures of IPV, such as the Composite Abuse Scale Revised Short Form [CAS_R_-SF] (Ford-Gilboe et al., 2016), are showing promise for disentangling patterns between and among men and women experiencing IPV in various types of relationships. To better align knowledge of experiences of IPV with measurement approaches, attention to survey items (and their applicability and fit for a range of diverse individuals) and how they are scored is critical (Tables 1 and 2).

**Table 1.** Summary of Critical Findings.


Men’s experiences of IPV victimization are poorly understood and understudied
What is known about men’s experiences of IPV highlights the importance of knowing the sex and gender of the perpetrator
Often researchers have focused on measuring incidents rather than identifying patterns; this approach has resulted in an incomplete understanding of violence and its prevalence

**Table 2.** Implications for Practice, Policy, and Research.


Understand that IPV is always gendered, and that there are patterns that are gendered, but those patterns are not adequately understood for people of all genders or for all types of sex and gender relations (e.g., opposite sex and same sex)
Develop and validate measures with people of all genders in relationships with people of all genders
Focus research on patterns of IPV in which the greatest harm occur
Conduct research to better understand factors that influence variation in men’s experiences (e.g., gender, sexual identify, and sex of the partner) and men’s vulnerability to, perceptions of, and responses to IPV.

Limitations associated with using only a sex-difference approach to understanding patterns of IPV can be extrapolated from two large population survey reports (Pottie-Bunge & Locke, 2000; Statistics Canada, 2016). Based on data disaggregated only according to the sex of the victim (male/female), similar rates of IPV were found between women and men. However, the reports highlighted substantive differences between men and women’s experiences of violence, reflective of key gender differences in patterns, severity and impact of IPV (e.g., women are more likely to report more severe forms of abuse and repeated victimization; men are less likely to be seriously injured and report fear that their lives are in danger; and women are more likely to report negative emotional consequences). These results highlight the importance of moving beyond comparison of incidences based only on sex of the victim to consider other factors known to influence experiences of IPV including sex and gender of the perpetrator. Thus, the ability to distinguish between subtypes of violence appears to be crucial to understanding differences that have traditionally been veiled by a gender-neutral orientation to identifying and measuring IPV in population surveys (H. Johnson, 2015) (For detailed reviews of the gender/sex symmetry debate see Bates & Graham-Kevan, 2016; Gilfus et al., 2010; Hines & Douglas, 2010; M. Johnson, 2011; H. Johnson, 2015).

In summary, emerging evidence related to patterns of violence based on sex-difference studies suggests that men are less likely than women to experience severe, frequent, and controlling IPV (H. Johnson, 2015; Public Health Agency of Statistics Canada, 2016) but are equally likely to experience less severe forms of IPV that can negatively affect health (Public Health Agency of Statistics Canada, 2016). Variation in men’s experiences of IPV exists, highlighting that some men are victims of more severe forms of IPV consistent with intimate terrorism, especially those in same-sex relationships. Collectively, this points to the need for caution in using scales/measures that only capture “occurrence” of various acts (yes/no) rather than capturing severity and looking for patterns of abuse/violence. Analyses must intentionally be contextualized to examine sex *and* gender. Simply counting isolated incidents could inflate prevalence rates while failing to recognize important distinctions between subtypes of violence and those groups experiencing the most severe forms of IPV; analyses that assess patterns of violence allow for more nuanced examination of differences *between* men and women as well as *among* men (and women). This has implications not only for how violence is measured, but also how data are scored and analyzed.

### Impact of Violence on Men’s Health

IPV has significant and harmful short- and long-term effects on health (Ansara & Hindin, 2011; Coker et al., 2005; Loxton, et al., 2017). Generally, isolated incidents of violence differ in their impact on health in comparison to more chronic, cumulative and severe violence, and the most damaging forms of IPV involve both physical aggression and isolation, intimidation, coercion/threats, and fear (Ansara & Hindin, 2011; Morgan and Wells, 2016; Scott-Storey, 2011). Further, it is well established that among men and women, a dose–response relationship exists, where greater severity of violence results in worse health outcomes (Holtzworth-Munroe, 2005; Scott-Storey, 2011; Scott-Storey et al., 2018).

Evidence of the pervasive and harmful impacts of IPV on men’s health is accumulating (Coker et al., 2002; Cook, 2009; Massetti et al., 2018). For example, data from the U.S. National Violence Against Women Survey (NVAWS) from 8001 men and 8005 women revealed that men who were victims of IPV experienced significant physical and mental health consequences (Coker et al., 2002). Similarly, Hines and Douglas’s (2015; 2018) studies in the US of 611 men seeking help for IPV and 1601 men from the general population showed that men who sought help for IPV reported significantly poorer mental health, including greater symptoms of post-traumatic stress disorder (PTSD) and depression. Other researchers have also reported increased rates of mental health problems such as anxiety, depression, PTSD, and suicidal ideation among men who experienced IPV (Chan et al., 2008; Hines, 2007; Hines & Douglas, 2010; Houry et al., 2008; Kaura & Lohman, 2007; Machado et al., 2018; Nybergh et al., 2016; Próspero, 2007; Randle & Graham, 2011; Stets & Straus, 1995; Tilbrook et al., 2010).

Additionally, men have reported physical injuries such as scratches, bruises, cuts, burns, broken bones, and stab and gunshot wounds as a result of IPV (Hines, 2015; Hines et al., 2007; McNeely et al., 2001; Nybergh et al., 2016). Researchers have noted that elevated blood pressure, sexually transmitted infections, asthma (Hines & Douglas, 2015), and somatic symptoms (Próspero, 2007) occur at higher rates among men who experience IPV than men in the general population. While existing research is limited, men in intimate relationships with other men report similar health-related IPV consequences to those reported by women in relationships with abusive men (Oliffe et al., 2014; Richards et al., 2003). Some men cope with stress and trauma through externalizing behaviors such as substance use, alcohol use, smoking, and antisocial behavior, all of which have the potential to negatively impact health (Black & Breiding, 2008; Carbone-Lopez et al., 2006; Coker et al., 2002; Comer, 1992; Douglas & Hines, 2011; Grant et al., 2004; Hines & Douglas, 2015; Massetti et al., 2018; Nybergh et al., 2016; O’Donnell et al., 2020; Randle & Graham, 2011; Rosenfield & Mouzon, 2013). Men have also reported giving up hobbies, missing work, loss of employment, and withdrawing from family and friends as a consequence of IPV (Carbone-Lopez et al., 2006; Entilli & Cipolletta. 2017; Gadd et al., 2003; Nybergh et al., 2016; Tilbrook et al., 2010). Thus, it is imperative to better understand variation *among* men and in particular whether certain patterns of violence result in more deleterious impact on men’s health and lives, allowing for better targeted interventions.

### Challenges to Inquiry

Men as victims of IPV remains controversial and understudied, despite empirical evidence documenting men’s exposure to, and experience of IPV dating back nearly five decades (e.g., Gelles, 1974). First, efforts to provoke meaningful social and policy responses to violence against women in intimate relationships led to dominance of the idea that IPV experienced by men is less frequent, severe, and consequential than IPV experienced by women (Dobash & Dobash, 2004; Douglas & Hines, 2011; Espinoza & Warner, 2016). This in turn contributed to an emphasis on research focused on women, with men studied primarily in comparison to women. However, such comparisons use women’s experiences as the starting point rather than fully understanding men’s experiences. Hines and Douglas (2009) have been particularly critical of the lack of research comparing abused and non-abused samples of men. They point out that findings from research comparing male victims to female victims has implied that men do not suffer to the same degree, which may erroneously trivialize men’s experiences of violence.

Differing theoretical perspectives about the nature of the IPV, inconsistent definitions of violence used, measures employed, and populations sampled (Anderson, 2005; Carvalho et al., 2011; Gilfus et al., 2010; Holtzworth-Munroe, 2005; Randle & Graham, 2011) has contributed to highly variable estimates of the prevalence and severity of IPV victimization in men. Researchers examining prevalence rates using population or national survey data often argue that “gender symmetry” or “sex symmetry” exists, implying that men and women are equally subjected to violence by an intimate partner (Straus, 2009; Tjaden & Thoennes, 2000). For example, *the Family Violence in Canada: A Statistical Profile* (2014) annual report documents that women and men equally reported physical and/or sexual violence by their partner during the preceding 5 years. While reports that men and women experience IPV at the same rate continue to be highlighted as evidence of “gender symmetry,” this is in fact a “sex-symmetry” argument. Simply comparing rates of IPV between women and men fails to take the gendered nature of IPV into account and is reflective of a continued and pervasive misunderstanding of gender. Many others have highlighted concerns related to sex and gender symmetry claims, citing inherent problems in the decontextualized way in which IPV is measured and consequently studied, especially among populations other than cis-gendered woman (Dragiewicz & DeKeseredy, 2012; H.; Johnson, 2015; M.; Johnson, 2006, 2011; McHugh, 2005).

### Direction for Future Inquiry

Despite the controversy and divergent results related to prevalence, it is clear that IPV affects *all people* regardless of their sex, gender or sexual orientation (Freedberg, 2006; Hines & Douglas, 2010). As such “partner violence must be addressed from a pragmatic and humanistic platform, upon which all suffering is a matter of concern and targeted with resolution” (Espinoza & Warner, 2016, p. 963). Importantly, the focus needs to shift away from the contentious gender/sex symmetry debate to dialogue about experiences and impacts of specifics subtypes and patterns of IPV among men, women, and people of all genders. Research is needed to better understand factors that influence variation in men’s experiences (e.g., gender, sexual identity, and sex/gender of the partner) and men’s vulnerability to, perceptions of, and responses to IPV.

Violence resulting in the greatest individual, social, and economic consequences should be of highest priority from a public health and social perspective. Thus, researchers should focus efforts on capturing the prevalence and consequences of IPV consistent with M. Johnson’s construct of intimate terrorism. Identifying and understanding the experiences of those most severely affected by violence regardless of sex and/or gender would serve as a foundation for informing public policy and initiating social change related to IPV (H. Johnson, 2015). As Kelly and Johnson (2008); Johnson (2008) state, “increased understanding and acceptance of differentiation among types of domestic violence by the broad spectrum of service providers, evaluators, academics, and policy makers will diminish the current turf and gender wars and lead to more effective partnerships and policies that share the common goal of reducing violence and its destructive effects on families” (p. 478).

However, the ability to adequately allocate support and resources to those who are most significantly affected by IPV (including men) depends on the accurate measurement of variation in experiences of IPV and an openness to identify such variation.

To obtain “accurate” prevalence rates of IPV, experiences of violence need to be measured in ways that capture context (examining severity, patterns, coercive control) and that consider the gendered aspects of the relationship as well as the context of the partnership (e.g., sex of the partner). Given that scales/measures are attached to gendered perspectives, it is imperative to identify the subtype(s) of IPV being measured (e.g., intimate terrorism and situational couple violence) and include indicators that reflect how that particular type of violence is experienced by people of all genders.

Validation of IPV measures for use among men is needed to demonstrate that they are appropriate, accurate, and comprehensive and that they reflect the construct of IPV as experienced by men (Follingstad & Rogers, 2013). This work has begun. For example, Machado et al. (2018) developed a survey to help contextualize men’s experience of IPV, their reactions to and the impacts of the violence. Scott-Storey et al. (2020) recently developed and tested the Cumulative Lifetime Violence Severity (CLVS) scale to measure perceived violence severity over the lifetime as a target and/or perpetrator; the scale includes questions measuring experiences of IPV and will contribute to an understanding of how cumulative lifetime violence, as well as how IPV in particular, affects men’s health and health outcomes.

Empirical studies to date suggest that for the most part, men identify and report similar acts of violence used against them as do women, and that much of the language used to describe acts of physical, psychological, sexual violence and coercive control resonates across genders and is consistent with the WHO definition of IPV. For example, Stephenson and Finneran (2013) developed a 30-item scale to measure IPV among gay and bisexual men based on data obtained from focus groups; with the exception of a few unique items (e.g., unintentionally transmit HIV to you; ask or tell you to “act straight” around certain people), the majority of the items were comparable to those seen on a multitude of IPV measures commonly used with women. This is significant as it provides beginning confidence that single scales/measures aimed at capturing IPV may be used across genders in prevalence studies and population surveys. The ability to differentiate gendered patterns and subtypes of violence is critical. Discerning whether individual acts of violence are part of a chronic pattern of aggressive control, induce fear, and result in harm to physical, mental, and social well-being should be the goal of future instrument development and analysis (Ansara & Hindin, 2011; Dragiewicz & DeKeseredy, 2012; Winstok, 2015).

## Conclusion

Understanding how men experience victimization from an abusive partner requires attention. While both men and women can be victims of IPV, the similarities and differences in their experiences are not adequately understood. Further, the gender and/or sex of the perpetrator shapes the experience of IPV. Although it appears that men experience similar “types” of IPV, there are differences in how these acts of violence are interpreted. As such, measuring IPV in the absence of context (e.g., meaning, severity, patterns, intention, gender, and sex of perpetrator) perpetuates the problem of false gender symmetry, obstructs accurate interpretation of results, and impedes comparisons across research studies (Follingstad & Rogers, 2013). Foremost, clarity is required regarding the desired construct being measured in IPV research; once this clarity is achieved, then measures and approaches will allow for more critical examination of the phenomenon, enabling a focus on variations across sex and gender categories. Collecting reliable, accurate data about the prevalence and magnitude of a social phenomenon such as IPV is of paramount importance to inform inclusive public policy and direct support and resources to those most affected. While much work still needs to be done, the current direction of the discussion is promising.
